# Facing Time in Ischemic Stroke: An Alternative Hypothesis for Collateral Failure

**DOI:** 10.1007/s00062-016-0507-2

**Published:** 2016-03-07

**Authors:** M. Pham, M. Bendszus

**Affiliations:** Department of Neuroradiology, Heidelberg University Hospital, INF 400, 69120 Heidelberg, Germany

**Keywords:** ischemic stroke, collaterals, acute therapy, endovascular recanalization, macrovascular, microvascular

## Abstract

Several randomized-controlled trials could recently demonstrate that ischemic stroke which is caused by large-cerebral-artery-occlusion can be treated effectively by endovascular recanalization. Among these studies, particularly the data from the ESCAPE study further corroborated the strong association between macrovascular pial collateral flow (before recanalization) and clinical outcome after recanalization. This review briefly gives an overview on these data and on the clinical key observations demonstrating this association in practice. Since the ischemic penumbra can only be sustained by collateral flow, the collapse of collateral blood flow or poor collateral filling, observed for example by DSA or CTA before recanalization, seems to be a primary cause of rapidly progressive infarction and futile therapeutic recanalization. However, it needs to be emphasized that the true cause-effect relationship between collateral failure and rapidly progressive infarction of the penumbra, i.e. the high probability of unfavorable clinical outcome despite recanalization, remains unclear. Along this line, an alternative hypothesis is offered viewing the collapse of collateral flow not as a cause but possibly as an inevitable secondary consequence of increasing peripheral/microvascular resistance during progressive infarction.

## Introduction

With the advance of highly effective endovascular treatment strategies [1–5] one further step in stroke treatment is to bring more patients within the reach of therapeutic recanalization. Hence, the future goal to further improve acute stroke therapy will have to be moved beyond the segment of arterial occlusion by aiming at the sustenance of the ischemic tissue-at-risk downstream (i.e., the penumbra) until recanalization can be achieved. This is highly desirable because with such a strategy at hand, additional time could be “bought” for the large number of patients in whom recanalization cannot be achieved fast enough by whatever type of intervention. In this regard, it is certainly among the most prominently discussed targets to increase the compensatory capacity of the pial (syn. leptomeningeal) collateral macrovasculature for maintaining sufficient blood flow into the ischemic penumbra [6]. Based on clinical key observations of collateral flow in acute ischemic stroke [3, 7], the pursuit of a therapeutic strategy which enhances macrovascular pial collateral blood flow seems reasonable and logical.

**Fig. 1:**
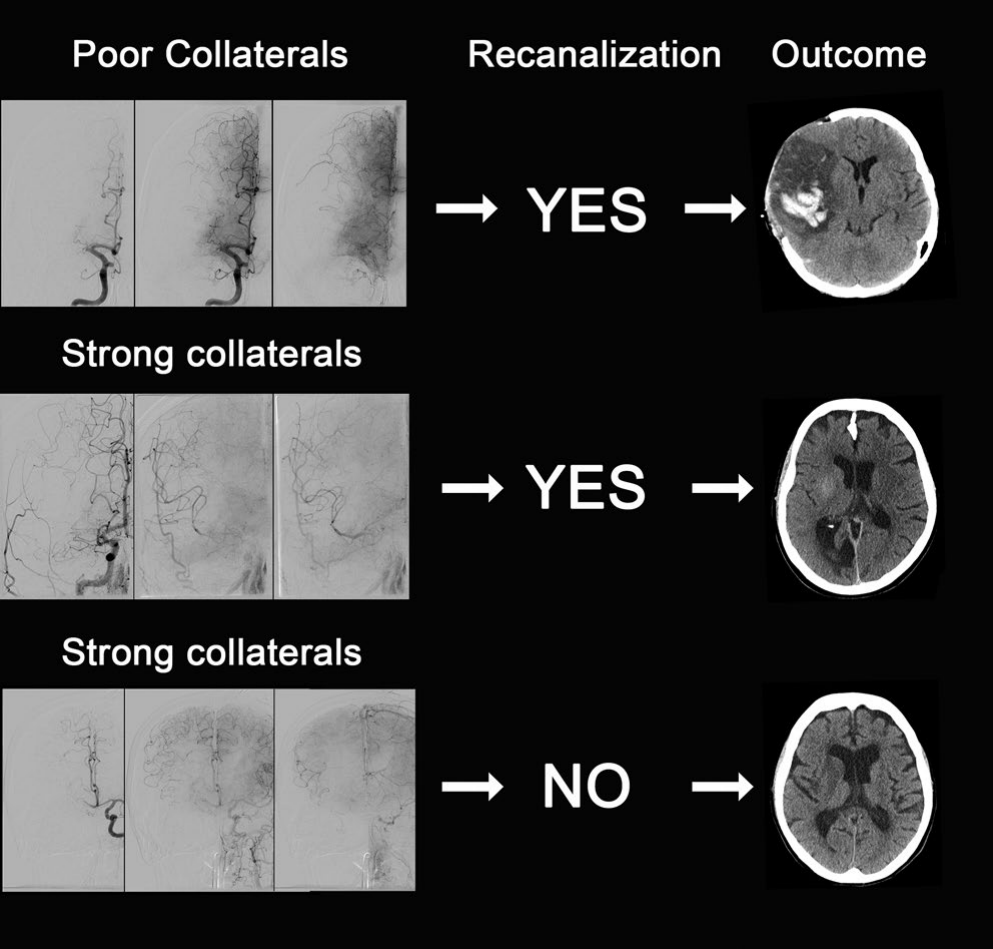
Strong association between collateral status at baseline and outcome. *Upper row from left to right*: Baseline frontal digital subtraction angiography (DSA) projection in arterial, parenchymal and early venous phase shows poor filling of the macrovascular pial arterial tree in right M1 occlusion. Despite successful recanalization (thrombolysis in cerebral infarction (TICI) 2b, not shown) unfavorable outcome with complete middle cerebral artery (MCA) infarction and hemorrhagic transformation ensued necessitating decompression hemicraniectomy (cranial computed tomography (CCT) on upper *right*). *Middle row*: Strong filling of pial collaterals at baseline in in right M1 occlusion. After successful recanalization (TICI 2b, not shown) favorable outcome was observed with only limited striatal infarction conspicuous by mild contrast extravasation. *Lower row*: Strong filling of pial collaterals at baseline in right internal carotid artery (ICA) occlusion (contralateral/*left* ICA injected). Even though recanalization could not be achieved in this case, neither by transfemoral access nor by direct carotid puncture, outcome was still favorable with only limited striatal infarction on follow-up CCT conspicuous by hypoattenuation (CCT on *lower right*)

These key observations can be summarized as follows:

1. Poor macrovascular pial collateral filling, as documented by computed tomography- (CT-), magnetic resonance- (MR-), or invasive digital subtraction angiography at baseline (before recanalization), is linked to poor outcome even if the occluded vessel segment is completely recanalized (a representative example of this observation is given in the upper row of Fig. 1).
2. Strong macrovascular pial collateral filling, as documented before recanalization, is linked to favorable outcome particularly if the occluded vessel segment can be recanalized successfully (a representative example of this observation is given in the middle row of Fig. 1).
3. Observations (1) and (2) need to be supplemented by the following clinical experience: Even if the occlusion persists (e.g., when recanalization fails for technical reasons) there is a certain, yet low number of patients who can achieve favorable outcome (with complete protection of the penumbra). However, this applies only if strong filling of the macrovascular pial arterial tree is present (a representative example of this observation is given in the lower row of Fig. 1).

This third observation has always impressed, and also relieved, caring clinicians and neuropathologists alike, starting with Charcot in 1882. He reported early that a major intracranial cerebral artery may be found occluded without the consequence of any structural cerebral injury downstream: “You must not think, gentlemen, that all obstructions of this kind would necessarily and inevitably produce effects so disastrous. There are instances, rare it is true, in which obstruction involving either a branch of the Sylvian artery [i.e. the middle-cerebral-artery] or the trunk of this vessel, in which such an occurrence may even remain without appreciable result.” [8].

Taking into consideration all these observations, the failure of macrovascular pial collaterals has been held responsible as a major *cause* of progressive cerebral infarction by many clinical and experimental researchers [9, 10]. This view strongly promotes that it seems particularly promising to enhance the structure and/or function of the pial collateral macrovasculature as a future therapeutic target.

In 2003, a very comprehensive review of this topic demonstrated, by reaching as far back as Heubner’s first observation of the macrovascular pial collaterals in 1872, that there has been an astonishingly regular periodicity of opposing trends and opinions either supporting or negating the view that the failure of the compensatory capacity of pial collaterals *causes* progressive infarction of the penumbra [9]. In the 1980s and 1990s of the past century, considerable reluctance prevailed to accept the failure of pial collaterals as a dominant factor in the progression of cerebral infarction [11–13]. Subsequently, another period arrived with its possible peak being in 2015 and 2016 when several randomized controlled trials (RCTs) proved strong treatment effects of endovascular recanalization in ischemic stroke caused by large vessel occlusion (e.g., of the M1 segment) [1–5]. Among these studies, particularly the findings of the ESCAPE study are highly relevant for research on the pial collateral macrovasculature [3].

In the The Endovascular Treatment for Small Core and Anterior Circulation Proximal Occlusion with Emphasis on Minimizing CT to Recanalization Times (ESCAPE) study, multiphase CT angiography was elegantly utilized to visualize with high structural resolution how the macrovascular pial arterial tree within the ischemic MCA field is filled by collateral backflow [3]. In this manner, the ESCAPE study arguably produced the best available data so far corroborating the strong association between robust collateralization and favorable outcome after recanalization. However, the cause–effect relationship between macrovascular pial collateral flow and outcome after recanalization may not be satisfactorily explained by the simple assumption that insufficient or declining pial collateral flow *causes* rapidly progressive infarction of the penumbra.

## Collateral failure: just a secondary consequence of progressive infarction?

This review summarizes clinical and experimental evidence which allows the alternative view that the cessation of blood flow through macrovascular pial collaterals may not be a dominant cause but only a secondary inevitable consequence of the progressive infarction of the penumbra. Accordingly, the collapse of blood flow in the pial collateral circulation may just be a macrovascular upstream (proximal) consequence of the pathological cellular and molecular events which occur further downstream (distally) within the ischemic microcirculation and which ultimately converge in microvascular failure. The two alternative possibilities of macrovascular failure preceding microvascular failure or vice versa may not mutually exclude each other completely. However, a separation seems necessary at least because future therapeutic targets need to be prioritized for experimental research and its clinical evaluation. In other words, it is reasonable to attempt to differentiate whether it is more promising to tackle the structural and/or functional capacity of pial collaterals upstream or, alternatively, to focus on the cellular and molecular mechanisms of ischemic microvascular failure downstream, or consider both as equally important targets.

One of the most convincing arguments denying that the pial collateral macrovasculature plays a dominant causative role in the progression of ischemic infarction recognizes vascular resistance as a key parameter. *Total cerebrovascular resistance* usually refers to the entire brain or an entire hemisphere owing to the fact that clinical and experimental methods of investigation can grasp this measure only on a global level. However, for the purpose of understanding the resistance properties of a certain compartment (e.g., the macro- vs. the microvasculature) or of a certain vascular segment (e.g., the pial collaterals), the perfusion path which leads into the specific vascular field/territory of interest needs to be deconstructed and considered in isolation [14]. Accordingly, the macrovascular bed is to be separated from the microvascular compartment if only the macrovascular contribution to resistance shall be considered. Now, if the isolated resistance of the macrovascular bed, under the physiological condition of an open middle-cerebral-artery (MCA) was similar to the isolated resistance of the collateral macrovascular perfusion path under the pathological condition of an M1 occlusion, then the macrovascular blood flow into the ischemic field downstream of the culprit occlusion should not critically depend on the macrovascular collateral channels, which connect the ischemic field with its neighboring vascular territories [9, 15]. In other words, if there was no or hardly any difference in the isolated macrovascular resistance between the normal/physiological and the collateral/pathological perfusion path, then the capacity of the collateral channels themselves should not be a limiting factor for blood flow into the ischemic field, and hence the pial collaterals should not influence the spatiotemporal dynamics of progressive penumbral infarction substantially. This argument is worth being considered further and needs to be explained in more detail.

## Hemodynamic consequences after M1 occlusion

Immediately after the abrupt embolic occlusion of a cerebral artery, blood flow behind the site of occlusion does not drop to zero as one might intuitively assume. Rather, it is maintained at a reduced level by residual flow distally to the occlusion [16]. Residual arterial inflow into macrovascular sites located distally to the occlusion is provided through a macrovascular network of collateral channels at the pial surface and also at the base of the brain. The collaterals relevant to the situation of an acute occlusion are represented by the pial macrovascular anastomoses with neighboring territories at the brain surface and/or by interconnected arterial segments at the base of the brain belonging to the circle of Willis. In the case of an occlusion of the M1 segment of the MCA, which is one of the most frequent culprit locations leading to severe ischemic stroke, residual flow distal to M1 depends mainly on the pial collateral and not the basal anastomoses of the circle of Willis. In such a situation, collateral flow circumventing the occlusion cannot be provided directly by the circle of Willis because its orifices lie proximally to the site of occlusion (M1). Therefore, in the event of an M1 occlusion, collateral flow into the ischemic M1 field is provided through the pial anastomoses between the ischemic and its neighboring territories of the anterior and posterior cerebral arteries located on the convex brain surface. Consequently, in an M1 occlusion pial collateral flow is provided relatively independent of the frequently occurring anatomical variations in the circle of Willis. For example, if the ipsilateral A1 segment is aplastic/hypoplastic, the contralateral A1 segment is typically dominant. It thus provides bilateral filling of both territories of the anterior cerebral artery (ACA; in humans and primates via the anterior communicating artery), and in this way provides pial collateral flow into the ipsilateral ischemic field (MCA territory) [17]. By contrast, if the ipsilateral A1 segment is dominant or codominant, pial collateral flow to the ipsilateral ischemic MCA territory comes via the ipsilateral A1 segment [17]. In both situations, substantial contributions may also come through the posterior cerebral artery (PCA) and its macrovascular pial anastomoses with the MCA territory mainly at its dorsal temporoparietal and temporooccipital edges.

The term “macrovascular pial” in this text refers to second-, third-, or fourth order branches of the major cerebral arteries on the brain surface and their macrovascular anastomoses connecting their respective terminal segments. For the MCA, these branches are commonly referred to as M2–M4 segments: M2 = the insular segments, M3 = the opercular segments and M4 = the terminal segments which overlie the convex brain surface [18]. A similarly useful and hierarchical nomenclature is established also for the anterior and posterior cerebral arteries. In a typical large-vessel occlusion, for example, of the M1 segment, its M2–M4 segments lie immediately distal (M2) to the site of occlusion as well as further distal on the opercular parts (M3) and the convex surface (M4) of the brain.

Those vascular structures that eventually carry the flowing arterial blood into the brain parenchyma are, however, not the macrovascular pial M2–M4 segments but a dense network of pial arterioles on the brain surface and the perforating arterioles, both of which mark the transition between the *macro-* and *micro-*circulation. The terminal macrovascular MCA segments (M4) typically span a size range of 250–1000 microns [19, 20], whereas the network of surface and penetrating arterioles typically ranges in diameter from 40 to 280 microns, according to the work of Duvernoy [21]. The “penetrating” or “perforating” arterioles branch off from the pial surface network. At the cortical surface these arterioles “perforate” the cortex and reach into the subcortex/white matter. At the base of the brain these perforators “perforate” the anterior perforate substance and reach into the base of the brain traversing (and supplying) white matter and deep grey matter (i.e., the basal ganglia). Immediately after an M1 occlusion, macrovascular blood flow can no longer reach the M2–M4 segments along the physiological path (via the open M1 segment) but has to arrive instead via collateral pathways connecting the ischemic MCA field (behind the occlusion) with the neighboring territories of the anterior and posterior cerebral arteries.

**Fig. 2:**
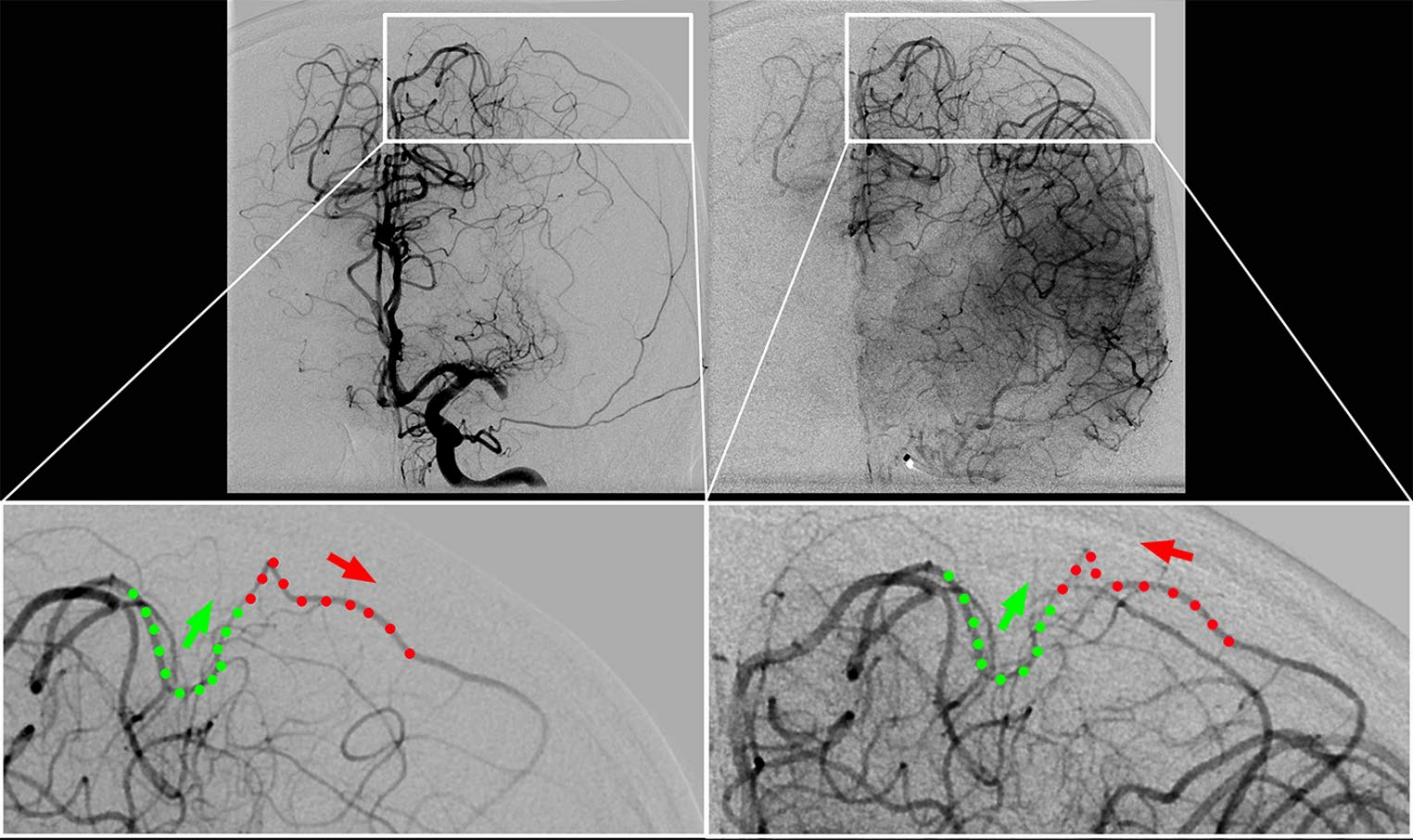
Macrovascular pial anastomosis between neighboring territories visualized by digital subtraction angiography (one exemplary perirolandic anastomosis between anterior cerebral artery (ACA) and middle cerebral artery (MCA) territory). *Upper left*: Left-sided occlusion of M1 segment (*left* internal carotid artery (ICA) Injection). The opacification in the arterial phase shows complete filling of ACA territory with far-reaching leptomeningeal terminal branches of the ACA arching over the brain convexity and backfilling terminal M4 segments of the ischemic MCA field. *Lower left (zoomed)*: The terminal ACA segment fills in antegrade fashion (*green dots* and *green arrow*), whereas the terminal M4 segment (*red dots* and *red arrow*) is filled in retrograde direction (from medial to lateral). *Upper right*: After complete endovascular recanalization of this left M1 occlusion, opacification in the late arterial phase shows a complete filling of ACA and MCA territories each in antegrade fashion, leaving the typical, physiological borderzone of perfusion between these territories. *Lower right (zoomed)*: The same terminal segments of the ACA *(green dots, green arrow)* and the MCA (*red dots, red arrow* now indicating reversal of flow from lateral to medial) are shown as before recanalization *(lower left)*. Now, after recanalization, the terminal M4 segment fills in the reverse, physiological direction (from lateral to medial), which is indicated by the reversed *red arrow*

These pial anastomoses are terminal macrovascular connections between the distal M4 branches on one side and the terminal macrovascular branches of either the ACA or PCA on the other side (Fig. 2). The structure of these pial macrovascular collateral channels had been described for the human brain by Heubner [22], Duret [23], Testut [24], Beevor [25], and others, and was then reported in 1953 with particular detail by vander Eecken [19]. Vander Eecken and coworkers counted and measured these anastomoses in human brains: Their average number between ACA ↔ MCA was 5.4 ± 1.7 (average diameter of 319 ± 87 μm) and 3.8 ± 0.9 between PCA ↔ MCA (average diameter of 362 ± 98 μm) [19]. Most of these channels are located in the depth of a sulcus and some overlie the gyral surface [19]. With modern fluoroscopy technology, including digital flat-panel detector DSA, which typically enables a pixel-by-pixel resolution of 100–200 microns [26], these anastomotic segments of the pial macrovasculature can be resolved. However, even with such exquisite structural resolution, the reliable visual recognition of the entirety of macrovascular collaterals remains difficult because of the complexity of the brain surface which hides many of these anastomoses behind multiple superimpositions. At least, the reliable visual detection of some of these anastomoses is possible (Fig. 2) and sometimes facilitated by sagittal projection. In a sagittal view, these macrovascular arterial segments span in a fan-like manner over the convex brain surface and appear as continuations of the terminal fourth order branches of the MCA connecting anteriorly (frontally), superiorly (frontoparietally), posteriorly and inferiorly (parietotemporally) with the terminal macrovascular branches of the ACA or PCA.

In healthy conditions, the anatomical–topographical position of these anastomoses is identical to the position of the physiological watershed zone of perfusion because this is where the distance to the neighboring source territories is greatest (e.g., the MCA field on the lateral side and ACA field on the medial side in antero–posterior view, Fig. 2). Obviously, the anatomical positions of these macrovascular anastomoses remain structurally fixed. The watershed zone of perfusion, however, is variable as a function of the balance in perfusion pressure between the neighboring territories. Therefore, under the pathological condition of an M1 occlusion, this watershed zone of perfusion moves away from the position of the collateral anastomoses towards the insula (moving across the convex brain surface from medial to lateral-inferior and into the Sylvian fissure). The insula is located just distal to the occluded M1 vessel segment and thus, under the condition of an M1 occlusion, represents the cortical region where the distance to the nourishing territories (ACA and PCA) is maximized (with blood flow from ACA/PCA passing through their collateral anastomoses with the M4 segments into the ischemic MCA field).

For this reason, the highest intensity of the perfusion deficit can be observed exactly in this functional end zone of M1 occlusion [27, 28], where the cortical core of infarction appears earliest and where it can be detected radiologically by early signs of infarction, for example, by CT as edema/hypoattenuation of the insular cortical ribbon [29, 30]. Cortical infarction typically progresses from the insular core over the opercular regions and into the convex brain surface areas of the frontoparietal, the superior, polar, and middle temporal cortex. This process occurs with variable spatial extension and with variable temporal dynamics which represents the variable recruitment of the penumbra (i.e., tissue-at-risk) by irreversible structural infarction. It is the aim of acute stroke therapy to protect or salvage the penumbra by fast recanalization and thereby to prevent that hypoxic-ischemic cell-death/infarction extends completely and spreads into the penumbra. Therapeutic recanalization typically does not aim at the early core of infarction since, in this region, ischemia is so profound that cell death has usually already occurred rather resembling instantaneous anoxic- than protracted hypoxic-ischemic cell death [31].

With the M1 segment occluded, the direction of flow in the pial collateral channels is reversed as compared to the physiological condition of an open M1 segment. In many other parts of the pial macrovascular M2–M4 arterial network, macrovascular blood flow distal to the occlusion will also be in reverse direction. Interestingly, however, the direction of flow in the ischemic field is not necessarily reversed everywhere (at all points of the macro- and microvascular network) contradicting what one would readily believe. This can be explained by the superficial, intracortical, and intra-subcortical three-dimensional structural complexity of the macro- and microvascular arterial network [32–35]. Thus, flow reversal occurs in only approximately 50 % of the pial macrovascular and in only 12 % of the pial arterioral surface network within the ischemic field [36, 37]. This becomes understandable when we consider the main direction of flow input into the cortical/subcortical microcirculation, which is provided through the perforating arterioles. In these precapillary vessels the direction of flow will be robustly antegrade, that is, from superficial to deeper layers irrespective of the direction of flow in the superficial vascular network, from which these perforators originate and receive flow input.

After an M1 occlusion, at those points which lie most distal from their source of supply the direction of macrovascular flow will be certainly reversed: Macrovascular blood flow in the M1 segment immediately behind the occlusion will be reversed and directed from lateral to medial (from M4/M3 into M2/M1). Conversely, under healthy conditions and after recanalization, it will be (re-) directed in the opposite direction, that is, from the terminal carotid and proximal M1 segment located medially to the distal M1 and M2–M4 segments located laterally (physiological/antegrade direction).

## Macrovascular resistance of the collateral perfusion path

As mentioned earlier, under the condition of an M1 occlusion, the macrovascular resistance of the collateral perfusion path (comprising the collateral channels of ACA ↔ MCA and PCA ↔ MCA) constitutes an important argument for seriously questioning if macrovascular collateral channels can play a dominant role at all in the progressive infarction of the penumbra.

According to Hagen–Poiseuille’s law, the most influential factor by far (with fourth power) determining macrovascular resistance is the total diameter of the macrovascular pial collateral path. On the basis of the most detailed morphometric anatomical data available so far [19], this total diameter can be approximated to 3–4 mm (average number of pial ACA ↔ MCA and PCA ↔ MCA anastomoses multiplied by their average diameter). Even in a subject with unfavorable variation of these channels with a total number and/or diameter in the lower range, the total diameter sum would still amount to approximately 2.5–3 mm. This would correspond very closely to the usual total distal diameter of the physiological perfusion path, which is represented by the M1 segment itself [18, 38]. Because of these similarities in the diameter sum, it can be assumed that the total macrovascular resistance against physiological unobstructed blood flow (i.e., along the usual path via an open M1 segment), should not be substantially different than for the pathological condition of obstructed flow (i.e., along the collateral path via the macrovascular pial anastomoses if the M1 segment is occluded).

Although certainly the dominant factor, total diameter obviously is not the only structural determinant of macrovascular resistance in Hagen–Poiseuille’s equation. The second *structural* factor determining the vascular resistance of the perfusion path is its (tube) length. The difference in path length between the two conditions (M1 open vs. occluded) at first glance may seem substantial and relevant. When estimated from the carotid-T, which represents the bifurcation where blood will either flow in the physiological direction (into the open M1 segment) or deflect into the pathological path (into the ipsilateral A1 segment if the M1 segment is occluded), the length of the physiological macrovascular path (from the carotid-T via M1 into M2 located at the center of the insula) in an adult human brain measures approximately 5 cm. By contrast, the length of the pathological parallel collateral paths measures approximately 20–25 cm: from the same starting point at the carotid-T into the A1 segment, moving around the genu of the corpus callosum, obliquely ascending in the midline from anterior to posterior and then arching over the convexity from medial to lateral, for example, in the perirolandic region, and finally flowing down (via a pial anastomosis) into a terminal M4 segment (and from there towards M3/M2). The length difference of around 15–20 cm between these two exemplary pathways (shorter unobstructed pathway via open M1 vs. longer collateral pathway under M1 occlusion) seems substantial, however, only in absolute terms. It seems negligible when contemplated in relative terms, that is, when the relation to the total length of the macrovascular resistance path, which must include also the much longer distance to the heart, is taken into account.

When Hagen–Poiseuille’s physical law is directly applied to the human physiology of blood rheology and hemodynamics, the functional properties of the vasculature and blood as a non-Newtonian fluid are neglected by making two simplifications: (1) Vessels are treated as rigid tubes with a constant diameter and tone, and (2) the viscosity of blood is assumed to be constant, which is largely true for blood plasma but not for the corpuscular fraction of blood. For the resistance properties of the macrocirculation under ischemic conditions, however, these two simplifications may be permissible and not represent oversimplifications for the following two reasons:

1. Functional alterations of the diameter of a vessel and/or the tone of the vascular wall are mediated by mechanisms of autoregulation. It can be assumed for severe ischemia that autoregulative mechanisms will likely be exhausted in the macro- and microcirculation with a consequence of maximum vasodilatation and loss of vascular tone [36]. Therefore, it seems unlikely for the regulation of vascular resistance that autoregulative mechanisms should dominate the structural capacity of the collateral circulation under the condition of ischemia.
2. With regard to the functional properties of blood it has to be stated that “with diminishing vessel diameter, the particulate nature of blood dominates the resistance to flow” [39]. Therefore, it can be expected that any alteration of blood viscosity must have its most pronounced effect on the microvascular resistance rather than on the macrovascular resistance. For this reason, it seems also unlikely for the regulation of vascular resistance that the functional viscous properties of blood itself dominate over the structural capacity of the collateral circulation, at least as far as the macrovascular path is concerned.

Blood viscosity is increased at the low flow velocities prevailing in the microcirculation and is decreased at the high flow velocities of the macrocirculation. Indeed, the viscosity of blood of acute stroke patients is further increased as could be shown by ex vivo viscometry [40]. Interestingly, this increase depends on blood flow velocity and is most marked at the low blood velocities/at low shear rates such as occurring in the microcirculation and not the macrocirculation [40]. Recently, it could be shown also in vivo that during low flow conditions such as during ischemia the apparent viscosity of blood increases [41]. This effect could be explained by the reduction of a so-called cell-free layer between streaming blood layers and the endothelium [41]. Under normal conditions of higher shear the corpuscular blood elements are forced away from the endothelium giving way for a cell-free layer which secures a decrease in apparent viscosity. At lower shear rates the thickness of the cell-free layer diminishes with the effect that apparent viscosity increases and with it microvascular resistance [41]. This modulation of viscosity represents a property not intrinsic to the structure or function of the microcirculation but to blood itself.

## Collapse of collateral flow secondary to microvascular failure?

For all these reasons, it seems unlikely that any structural or functional limitation of the collateral *macro-*circulation should play a dominant role in the progression of ischemic infarction. Instead, it appears more plausible that structural and/or functional properties of the *micro-*circulation crucially determine total vascular resistance during ischemia and, as a consequence thereof also govern cerebral blood flow through the upstream collateral macrovascular pathways. Obviously, apart from vascular resistance, the pressure drop across the total vascular bed is the other force which drives (collateral) flow. Indeed, the close guidance of systemic arterial pressure to maintain cerebral perfusion pressure under ischemia is an obvious and already well-exhausted therapeutic strategy before and during recanalization therapy. However, no therapeutic strategy exists to date which aims at the cellular and molecular mechanisms converging in microvascular failure. This failure is ultimately associated with an excessive increase of microvascular resistance and finally complete microvascular obstruction.

As in other organs, also the total vascular resistance of the brain (cerebrovascular resistance, CVR) under healthy conditions but even more so during ischemia is heavily dominated by the microcirculation. It could be estimated that the largest part of the total or regional CVR (the term “regional” referring to a certain vascular field) under healthy conditions is determined by the pial arterioles [36]. Another early experimental finding strongly underlines that the CVR is mainly governed by the resistance of the microcirculation to a much larger degree than by any contribution of macrovascular segments upstream. Through costly and invasive experimental work, Bakay and Sweet already showed in 1952 that only 20 % of the mean systemic arterial pressure is needed to drive blood through the macrovascular segments of the systemic and cerebral arterial circulation [42]. It follows that the biggest share of the perfusion pressure (drop) needs to be spent to overcome the downstream resistance of the microcirculation.

Overwhelming experimental evidence on the cellular and molecular level indicates why microvascular resistance increases excessively during ischemia. Starting with the early work of Ames et al. it became obvious that swelling of the microvascular endothelium in the ischemic brain and of the perimicrovascular glia causes a significant narrowing of the microvascular lumen. If ischemia is severe and endures long enough even complete microvascular obstruction ensues—this was coined the “no-reflow” phenomenon [43]. This denomination implies that under certain unfavorable circumstances effective reperfusion of the microcirculation may not occur even if proximal/macrovascular recanalization is successfully achieved [43].

By the same group another important observation was made which was already referred to above: The viscosity of patient blood obtained early after acute ischemic stroke is particularly increased at low shear rates, which clearly points to the fact that resistance mainly comes into effect at the level of the microcirculation [44]. These authors were among the first to hypothesize that “changes in the blood itself”, possibly through aggregation of its corpuscular/cellular elements, were responsible for the increase of viscosity at low shear rates occurring predominantly in the *micro*- and not the *macro*-circulation [44]. In subsequent decades it could be clearly shown that blood cell-to-cell and blood cell-to-endothelium as well as blood-cell-to-subendothelium interactions (reviewed in detail, e.g., by del Zoppo in 2003 [45] and by De Meyer in 2016 [46]) lead to microvascular failure and may result in a complete obstruction of the microcirculation thereby excessively raising the microvascular resistance. It is now firmly established that endothelial adhesion of white blood cells [47–49], endothelial adhesion of platelets [50–52], and ultimately thrombus formation involving also the intrinsic coagulation system [53] are mechanisms, which are critical for the progressive infarction of the penumbra and which all operate on the level of the ischemic microcirculation. Moreover, a complex molecular and cellular interaction between thrombotic and inflammatory pathways has been implied recently suggesting that the ischemic degeneration of the microvasculature and of the adjacent neuronal and glial brain substance may be a joint result of microvascular thrombosis and inflammatory mechanisms jointly called "thromboinflammation" [46, 54–56]. The structural/functional final result of these events is the complete luminal occlusion of the ischemic microcirculation.

In consistence with these mechanisms, a myriad of cellular and molecular targets have been identified, which can already be used effectively to alleviate, decelerate, or destabilize microvascular obstruction [57]. As a consequence, it has become possible to achieve a significant prolongation of the time window for efficient recanalization and inhibit infarct expansion within the penumbra, however, only under experimental conditions. In contrast to the high number of experimentally effective targets, so far, not a single of these strategies could be made available for human use because clinical efficacy could be demonstrated in none of the clinical studies including phase II and III trials [31, 57]. As a result of this discrepancy between bench and bedside, it has always been called into question if the most widely used rodent model, the transient occlusion of the MCA, could ever reflect human stroke appropriately [31]. Another explanation seems possible as well but has found clearly less consideration: In human stroke the therapeutic effectiveness of macrovascular recanalization did just not reflect the experimental situation well enough. In a typical experimental setting, the occlusive stimulus is simply removed after a defined time interval to study how the progressive infarction of the penumbra can be delayed. Such a complete control of macrovascular recanalization is not achievable in the human setting. For example, the recanalization rates of only intravenous (IV) thrombolysis are low. The best available data in this regard came from early phase II thrombolysis trials documenting by gold-standard invasive angiography poor recanalization rates of only 8 % for ICA, 26 % for M1, and 35 % for M2 occlusions [58]. By comparison, in the widespread thread occlusion models of embolic stroke in rodents, for obvious reasons, a near-perfect control of the recanalization rate will be achievable certainly being close to 100 % (modelling ICA/M1 occlusion). Beyond the insufficient human recanalization rates of the past, other substantial differences exist between the clinical and experimental condition. In human stroke, subocclusive thrombembolic partial occlusion is not a rare event, and it is typically not simulated by experimental research. Furthermore, thrombus fragmentation with distal embolization either occurring spontaneously or during therapeutic intervention by mechanical thrombectomy and/or by pharmacological thrombolysis is not accounted for in the experimental situation.

To prolong the viability of and finally salvage the penumbra, or at least clinically significant parts of it, with strategies focusing on the ischemic microcirculation, may require timely and complete macrovascular recanalization as a sine qua non condition that has to be established in conjunction. For large-vessel macrovascular occlusions, which are typically modelled by experimental embolic stroke, only recently, the therapeutic recanalization rates in humans could be increased substantially by endovascular thrombectomy, approaching or even exceeding a rate of 80% of complete recanalization followed by strongly positive clinical effects [1-5]. with strong clinical effects [1–5]. Such an improvement of recanalization rates in humans may only now facilitate the breakthrough of adjunctive strategies that prevent or reverse the molecular and cellular events which lead to downstream microvascular failure and an increase of resistance.

The angiographic data from several endovascular stroke trials has enabled another noteworthy observation also centering on the role of the macrovascular pial collateral circulation in acute ischemic stroke [59, 60]. Poor collateral flow at baseline (before recanalization) is strongly predictive of poor recanalization/reperfusion itself [59, 60]: It was observed that lower recanalization/reperfusion grades as scored by the modified thrombolysis in cerebral infarction (TICI) scale were closely associated with poorer collaterals *before* recanalization and that, by contrast, higher/better grades of TICI (in particular 2b and 3) were much more likely to occur in the case of strong collaterals *before* recanalization. How can it be explained that the patency and antegrade filling in segments located distally to the occluded vessel segment *after* its recanalization should depend on the degree and robustness of their retrograde collateral filling *before* recanalization? Distal embolization has been offered as one explanation; however, it would remain difficult to explain why, particularly at the moment of antegrade flow reversal/restoration, distal embolization should occur more frequently in patients with poor retrograde collateral filling (before recanalization). Enhanced transportation of intravenously administered thrombolytics to the ischemic field via the collateral pathways preventing secondary occlusions in the downstream pial arterial tree was also offered and cannot be refuted [6]. Yet another explanation seems plausible which also takes into account the probable hemodynamic and rheologic effects that the distal ischemic microcirculation has on the proximal pial macrovasculature. Despite successful local recanalization of the proximal culprit occlusion, distal filling defects or stasis filling responsible for TICI 2a or worse could merely be an effect of an increased regional microvascular resistance during progressive infarction. This explanation is supported at least by evidence from the macro- and microvasculature of the heart during ischemia/reperfusion.

Similar to the angiographic appearance of unfavorable TICI scores (stasis filling and/or filling defects in the pial macrovasculature distal to the culprit occlusion), worse TIMI flow grades in the heart have been clearly linked to alterations in microvascular resistance [61, 62]. In the epicardial circulation, this link between macro- and microvasculature has not only been established by reasoning but also by functional hemodynamic invasive measurements (i.e., empirical indices of microvascular resistance) [63–65], which are not available for the pial macrovasculature in humans.

It can be summarized that after the advance of highly effective recanalization strategies, the ultimate goal in stroke treatment now becomes to gain more time for the large number of patients in whom recanalization cannot be provided fast enough. To sustain and prolong the viability of the ischemic tissue downstream of the culprit macrovascular occlusion (i.e., of the penumbra) would “buy” more time for therapeutic recanalization by endovascular mechanical, intravenous thrombolytic, or both means. Interindividual differences in the spatial extension and in the temporal dynamics of progressive penumbral infarction, and thus, in the potential to achieve a favorable clinical response to therapeutic recanalization could be clearly associated with salient interindividual characteristics of collateral macrovascular blood flow reaching the ischemic field through pial collateral channels/anastomoses (before recanalization). Such a strong association suggests but cannot prove causality (between interindividual differences in the structural and/or functional properties of the macrovascular collateral channels and the notorious clinical differences in response after recanalization). There exist other reasons to believe that poor collateral backfilling before recanalization is not the *cause* of unfavorably fast progressive infarction or lack of response to recanalization. Poor collateral backfilling of the pial macrovasculature may just be a *secondary consequence* of increasing peripheral/distal (micro-)vascular resistance which may be inevitably caused during progressive infarction by molecular and cellular mechanisms well-known by experimental investigation to compromise microvascular function during ischemia/reperfusion during ischemia/reperfusion. In addition, human data on the anatomical structure of the macrovascular collateral anastomoses and on the altered viscous properties of blood during ischemic stroke suggest that crucial interindividual differences might exist with regard to the resistance properties of the micro- rather than the pial macrocirculation. In the future, the question whether it is macro- or microvascular failure or both that drives the progressive infarction of the penumbra will be crucial. To be able to address this question will depend on the development of methods that enable the measurement of cerebrovascular resistance during ischemia and after recanalization. However, we can see particularly in the past work from Hossmann and coworkers, that a careful and very elaborate experimental design has to be undertaken to be able to differentiate the contributions of different compartments or vascular segments to the total resistance of a certain vascular region [14]. These experimental methods obviously are not readily applicable in the clinical situation. Therefore, the further development of radiological imaging methods such as CT- or MR-perfusion needs to be challenged to finally answer if, also in human stroke, microvascular failure and increased resistance play an equally substantial role as in the experimental setting. In fact, these noninvasive imaging techniques already deliver perfusion information on a tissue-/microvascular level but it is still not possible to differentiate between the micro- and macrovascular contribution to the respective perfusion contrast.

## References

[1] Berkhemer OA, Fransen PS, Beumer D, van den Berg LA, Lingsma HF, Yoo AJ (2015). A randomized trial of intraarterial treatment for acute ischemic stroke. N Engl J Med.

[2] Campbell BC, Mitchell PJ, Kleinig TJ, Dewey HM, Churilov L, Yassi N (2015). Endovascular therapy for ischemic stroke with perfusion-imaging selection. N Engl J Med.

[3] Goyal M, Demchuk AM, Menon BK, Eesa M, Rempel JL, Thornton J (2015). Randomized assessment of rapid endovascular treatment of ischemic stroke. N Engl J Med.

[4] Jovin TG, Chamorro A, Cobo E, de Miquel MA, Molina CA, Rovira A (2015). Thrombectomy within 8 h after symptom onset in ischemic stroke. N Engl J Med.

[5] Saver JL, Goyal M, Bonafe A, Diener HC, Levy EI, Pereira VM (2015). Solitaire with the intention for thrombectomy as primary endovascular treatment for acute ischemic stroke (SWIFT PRIME) trial: protocol for a randomized, controlled, multicenter study comparing the Solitaire revascularization device with IV tPA with IV tPA alone in acute ischemic stroke. Int J Stroke.

[6] Liebeskind DS (2003). Collateral circulation. Stroke.

[7] Leng X, Fang H, Leung TW, Mao C, Miao Z, Liu L (2015). Impact of collaterals on the efficacy and safety of endovascular treatment in acute ischaemic stroke: a systematic review and meta-analysis. J Neurol Neurosurg Psychiatry.

[8] Charcot JM (1884). Arterial circulation of the cerebrum (Lectures V and VI). Lectures on the localization of cerebral and spinal diseases.

[9] Brozici M, van der Zwan A, Hillen B (2003). Anatomy and functionality of leptomeningeal anastomoses: a review. Stroke.

[10] Sheth SA, Sanossian N, Hao Q, Starkman S, Ali LK, Kim D (2016). Collateral flow as causative of good outcomes in endovascular stroke therapy. J Neurointerv Surg.

[11] Derdeyn CP, Powers WJ, Grubb RL (1998). Hemodynamic effects of middle cerebral artery stenosis and occlusion. AJNR Am J Neuroradiol.

[12] Aaslid R (1999). Hemodynamics of cerebrovascular spasm. Acta Neurochir Suppl.

[13] Fieschi C, Argentino C, Lenzi GL, Sacchetti ML, Toni D, Bozzao L (1989). Clinical and instrumental evaluation of patients with ischemic stroke within the first six hours. J Neurol Sci.

[14] Date H, Hossmann KA (1984). Effect of vasodilating drugs on intracortical and extracortical vascular resistance following middle cerebral artery occlusion in cats. Ann Neurol.

[15] Fields WS (1972). Collateral circulation in cerebrovascular disease. Handbook of Clinical Neurology.

[16] Symon L, Ishikawa S, Meyer JS (1963). Cerebral arterial pressure changes and development of leptomeningeal collateral circulation. Neurology.

[17] Liebeskind DS, Flint AC, Budzik RF, Xiang B, Smith WS, Duckwiler GR (2015). Carotid Iʼs, Lʼs and Tʼs: collaterals shape the outcome of intracranial carotid occlusion in acute ischemic stroke. J Neurointerv Surg.

[18] Krayenbühl H, Yasargil MG, Huber P (1982). Cerebral Angiography.

[19] Vander Eecken HM, Adams RD (1953). The anatomy and functional significance of the meningeal arterial anastomoses of the human brain. J Neuropathol Exp Neurol.

[20] Kadri PA, Krisht AF, Gandhi GK (2007). An anatomic mathematical measurement to find an adequate recipient M4 branch for superficial temporal artery to middle cerebral artery bypass surgery. Neurosurgery.

[21] Duvernoy HM, Delon S, Vannson JL (1981). Cortical blood vessels of the human brain. Brain Res Bull.

[22] Heubner O (1874). Die luetischen Erkrankungen der Hirnarterien.

[23] Duret H (1874). Recherches anatomiques sur la circulation de l’encephale. Arch Phisiol Norm Pathol.

[24] Testut JL (1895). Traité d’anatomie humaine. Anatomie descriptive-histologie-développment.

[25] Beevor CE (1909). On the distribution of different arteries supplying the human brain. Philos Trans R Soc Lond B Biol Sci.

[26] Kalender WA, Kyriakou Y (2007). Flat-detector computed tomography (FD-CT). Eur Radiol.

[27] Symon L, Branston NM, Strong AJ, Hope TD (1977). The concepts of thresholds of ischaemia in relation to brain structure and function. J Clin Pathol Suppl (R Coll Pathol).

[28] Symon L, Lassen NA, Astrup J, Branston NM (1977). Thresholds of ischaemia in brain cortex. Adv Exp Med Biol.

[29] von Kummer R, Allen KL, Holle R, Bozzao L, Bastianello S, Manelfe C (1997). Acute stroke: usefulness of early CT findings before thrombolytic therapy. Radiology.

[30] von Kummer R, Bourquain H, Bastianello S, Bozzao L, Manelfe C, Meier D (2001). Early prediction of irreversible brain damage after ischemic stroke at CT. Radiology.

[31] Hossmann KA (2012). The two pathophysiologies of focal brain ischemia: implications for translational stroke research. J Cereb Blood Flow Metab.

[32] Shih AY, Driscoll JD, Drew PJ, Nishimura N, Schaffer CB, Kleinfeld D (2012). Two-photon microscopy as a tool to study blood flow and neurovascular coupling in the rodent brain. J Cereb Blood Flow Metab.

[33] Nishimura N, Schaffer CB, Friedman B, Lyden PD, Kleinfeld D (2007). Penetrating arterioles are a bottleneck in the perfusion of neocortex. Proc Natl Acad Sci U S A.

[34] Guibert R, Fonta C, Risser L, Plouraboue F (2012). Coupling and robustness of intra-cortical vascular territories. Neuroimage.

[35] Blinder P, Tsai PS, Kaufhold JP, Knutsen PM, Suhl H, Kleinfeld D (2013). The cortical angiome: an interconnected vascular network with noncolumnar patterns of blood flow. Nat Neurosci.

[36] Shih AY, Friedman B, Drew PJ, Tsai PS, Lyden PD, Kleinfeld D (2009). Active dilation of penetrating arterioles restores red blood cell flux to penumbral neocortex after focal stroke. J Cereb Blood Flow Metab.

[37] Schaffer CB, Friedman B, Nishimura N, Schroeder LF, Tsai PS, Ebner FF (2006). Two-photon imaging of cortical surface microvessels reveals a robust redistribution in blood flow after vascular occlusion. PLoS Biol.

[38] Schreiber SJ, Gottschalk S, Weih M, Villringer A, Valdueza JM (2000). Assessment of blood flow velocity and diameter of the middle cerebral artery during the acetazolamide provocation test by use of transcranial Doppler sonography and MR imaging. AJNR Am J Neuroradiol.

[39] Lipowsky HH (2005). Microvascular rheology and hemodynamics. Microcirculation.

[40] Ott EO, Lechner H, Aranibar A (1974). High blood viscosity syndrome in cerebral infarction. Stroke.

[41] Yalcin O, Ortiz D, Williams AT, Johnson PC, Cabrales P (2015). Perfusion pressure and blood flow determine microvascular apparent viscosity. Exp Physiol.

[42] Bakay L, Sweet WH (1952). Cervical and intracranial intra-arterial pressures with and without vascular occlusion. Surg Gynecol Obstet.

[43] Ames A, Wright RL, Kowada M, Thurston JM, Majno G (1968). Cerebral ischemia. II. The no-reflow phenomenon. Am J Pathol.

[44] Fischer EG, Ames A, Lorenzo AV (1979). Cerebral blood flow immediately following brief circulatory stasis. Stroke.

[45] del Zoppo GJ, Mabuchi T (2003). Cerebral microvessel responses to focal ischemia. J Cereb Blood Flow Metab.

[46] De Meyer SF, Denorme F, Langhauser F, Geuss E, Fluri F, Kleinschnitz C (2016). Thromboinflammation in stroke brain damage. Stroke.

[47] Okada Y, Copeland BR, Fitridge R, Koziol JA, del Zoppo GJ (1994). Fibrin contributes to microvascular obstructions and parenchymal changes during early focal cerebral ischemia and reperfusion. Stroke.

[48] Okada Y, Copeland BR, Mori E, Tung MM, Thomas WS, del Zoppo GJ (1994). P-selectin and intercellular adhesion molecule-1 expression after focal brain ischemia and reperfusion. Stroke.

[49] Connolly ES, Winfree CJ, Springer TA, Naka Y, Liao H, Yan SD (1996). Cerebral protection in homozygous null ICAM-1 mice after middle cerebral artery occlusion. Role of neutrophil adhesion in the pathogenesis of stroke. J Clin Invest.

[50] Choudhri TF, Hoh BL, Zerwes HG, Prestigiacomo CJ, Kim SC, Connolly ES (1998). Reduced microvascular thrombosis and improved outcome in acute murine stroke by inhibiting GP IIb/IIIa receptor-mediated platelet aggregation. J Clin Invest.

[51] Kleinschnitz C, Pozgajova M, Pham M, Bendszus M, Nieswandt B, Stoll G (2007). Targeting platelets in acute experimental stroke: impact of glycoprotein Ib, VI, and IIb/IIIa blockade on infarct size, functional outcome, and intracranial bleeding. Circulation.

[52] Kleinschnitz C, De Meyer SF, Schwarz T, Austinat M, Vanhoorelbeke K, Nieswandt B (2009). Deficiency of von Willebrand factor protects mice from ischemic stroke. Blood.

[53] Kleinschnitz C, Stoll G, Bendszus M, Schuh K, Pauer HU, Burfeind P (2006). Targeting coagulation factor XII provides protection from pathological thrombosis in cerebral ischemia without interfering with hemostasis. J Exp Med.

[54] Gob E, Reymann S, Langhauser F, Schuhmann MK, Kraft P, Thielmann I (2015). Blocking of plasma kallikrein ameliorates stroke by reducing thromboinflammation. Ann Neurol.

[55] Stoll G, Kleinschnitz C, Nieswandt B (2010). Combating innate inflammation: a new paradigm for acute treatment of stroke?. Ann N Y Acad Sci.

[56] Nieswandt B, Kleinschnitz C, Stoll G (2011). Ischaemic stroke: a thrombo-inflammatory disease?. J Physiol.

[57] O’Collins VE, Macleod MR, Donnan GA, Horky LL, van der Worp BH, Howells DW (2006). 1,026 experimental treatments in acute stroke. Ann Neurol.

[58] del Zoppo GJ, Poeck K, Pessin MS, Wolpert SM, Furlan AJ, Ferbert A (1992). Recombinant tissue plasminogen activator in acute thrombotic and embolic stroke. Ann Neurol.

[59] Liebeskind DS, Tomsick TA, Foster LD, Yeatts SD, Carrozzella J, Demchuk AM (2014). Collaterals at angiography and outcomes in the Interventional Management of Stroke (IMS) III trial. Stroke.

[60] Liebeskind DS (2014). Collateral lessons from recent acute ischemic stroke trials. Neurol Res.

[61] Ito H, Okamura A, Iwakura K, Masuyama T, Hori M, Takiuchi S (1996). Myocardial perfusion patterns related to thrombolysis in myocardial infarction perfusion grades after coronary angioplasty in patients with acute anterior wall myocardial infarction. Circulation.

[62] Ito H, Maruyama A, Iwakura K, Takiuchi S, Masuyama T, Hori M (1996). Clinical implications of the ’no reflow’ phenomenon. A predictor of complications and left ventricular remodeling in reperfused anterior wall myocardial infarction. Circulation.

[63] Ambrosio G, Savino K (2014). CMR assessment of microvascular obstruction in STEMI: ready for prime time?. J Am Coll Cardiol.

[64] Cuculi F, De Maria GL, Meier P, Dall’Armellina E, de Caterina AR, Channon KM (2014). Impact of microvascular obstruction on the assessment of coronary flow reserve, index of microcirculatory resistance, and fractional flow reserve after ST-segment elevation myocardial infarction. J Am Coll Cardiol.

[65] Wu KC, Zerhouni EA, Judd RM, Lugo-Olivieri CH, Barouch LA, Schulman SP (1998). Prognostic significance of microvascular obstruction by magnetic resonance imaging in patients with acute myocardial infarction. Circulation.

